# The impact of cardiogenic shock and out-of-hospital cardiac arrest on the outcome of acute myocardial infarction: a national-level analysis

**DOI:** 10.1007/s11739-025-03984-6

**Published:** 2025-06-13

**Authors:** Pavol Tomasov, Zuzana Motovska, Ota Hlinomaz, Petr Kala, Marek Sramko, Jan Mrozek, Milan Hromadka, Jan Precek, Josef Bis, Jan Matejka, Tamilla Muzafarova, Pavel Cervinka, Ales Kovarik, Libor Sknouril, Zdenek Coufal, Jiri Jarkovsky

**Affiliations:** 1 Cardiocenter, Liberec Regional Hospital, Liberec, Czech Republic; 2 Cardiocentre, Third Faculty of Medicine, Charles University and University Hospital Kralovske Vinohrady, Srobarova 50, 100 34 Prague, Czech Republic; 3 First Department of Internal Medicine and Cardioangiology, International Clinical Research Center, Faculty of Medicine, St. Anne’s University Hospital, Masaryk University, Brno, Czech Republic; 4 Department of Internal Medicine and Cardiology, University Hospital, Faculty of Medicine of Masaryk University, Brno, Czech Republic; 5 Institute of Clinical and Experimental Medicine, Cardiology, Prague, Czech Republic; 6 Department of Cardiovascular Surgery, University Hospital, Ostrava, Czech Republic; 7 Department of Cardiology, Faculty of Medicine, University Hospital, Charles University, Pilsen, Czech Republic; 8 Department of Internal Medicine I and Cardiology, Faculty of Medicine and Dentistry, University Hospital, Palacky University, Olomouc, Czech Republic; 9 Departmet of Cardiovascular Medicine, University Hospital, Hradec Kralove, Czech Republic; 10 Department of Cardiology, Hospital of Pardubice, Pardubice, Czech Republic; 11 Department of Cardiology, Krajska Zdravotni A.S., Masaryk Hospital, Jan Evangelista Purkyne University, Usti nad Labem, Czech Republic; 12 Regional Hospital, Ceske Budejovice, Czech Republic; 13 Department of Cardiology, Nemocnice Agel, Trinec-Podlesi, Trinec, Czech Republic; 14 Department of Cardiology, T. Bata Regional Hospital, Zlin, Czech Republic; 15 Institute of Health Information and Statistics of the Czech Republic, Prague, Czech Republic; 16 Institute of Biostatistics and Analyses, Faculty of Medicine, Masaryk University, Brno, Czech Republic

**Keywords:** Acute myocardial infarction, Out-of-hospital cardiac arrest, Cardiogenic shock, Outcome, Predictors

## Abstract

Cardiogenic shock (CS) and out-of-hospital cardiac arrest (OHCA) are events with profound implications for patient outcomes. We aim to analyze the predictors of CS and OHCA in patients with acute myocardial infarction and their effects on mortality. The analysis is based on data from a national registry between 2016 and 2020. A total of 23,703 patients with ST-elevation myocardial infarction (STEMI) were analyzed: (A) patients without CS and OHCA (19,590), (B) after OHCA (2,262), (C) with CS (713), and (D) after OHCA with CS (1,138). Patients after OHCA without CS had the lowest mean age [62.0 (± 12.6) years], while patients with CS without OHCA were the oldest [68.8 (± 11.8) years] and had the highest proportions of comorbidities. CS was a predictor of 30-day and 1-year mortality, with odds ratios [OR; 95% confidence intervals (CI)] of 5.52 (4.51; 6.75) and 4.66 (3.87; 5.61) for patients after OHCA, and OR (95% CI) 9.28 (7.56; 11.38) and 7.33 (6.04; 8.89) for those without OHCA. For overall survival up to 30 days and in comparison to patients without CS and OHCA, the hazard ratios (95% CI) was 2.77 (2.40; 3.20) for patients with OHCA only, 14.36 (12.57; 16.40) for patients with CS only, and 16.96 (15.19; 18.92) for patients with both CS and OHCA. OHCA altered the 30-day mortality risk after STEMI for both patients with and without CS. CS is a predictor of both 30-day and 1-year mortality in patients with STEMI, irrespective of OHCA status.

## Introduction

Cardiogenic shock (CS) and out-of-hospital cardiac arrest (OHCA) are critical cardiovascular events with profound implications for patient outcomes. Acute myocardial infarction (AMI) remains a leading cause of both CS and OHCA [1]. There is limited evidence regarding treatment strategies in CS, which presents a challenge in contemporary cardiology and emergency medicine [2, 3]. A better understanding of epidemiology, risk factors, and prognostic implications of CS and OHCA could provide insights, eventually leading to improved patient care.

There has been an upward trend in the number of AMIs complicated by CS and OHCA [4–7], which both increase the mortality of patients with AMI [2, 8–10]. Cardiogenic shock has been shown to complicate up to 10% of AMIs [3, 11]; moreover, despite some decline in mortality rates in registry data, randomized controlled trials (RCTs) reveal 30-day mortality rates as high as 40–60% [5, 9, 10, 12]. By comparison, the prevalence of OHCA in AMI patients treated with primary percutaneous coronary intervention (PCI) varies greatly, but can reach 30% in specific settings, with 30-day mortality rates exceeding 35% [13–16].

When OHCA occurs in AMI patients, it shifts the most frequent cause of death from primary cardiac reasons to neurological injury, outweighing the cardiac risks in the overall AMI population [6]. The population of AMI patients with OHCA admitted to hospital is heterogeneous and differs depending on the setting of prehospital care or the rules for the termination of cardiopulmonary resuscitation [13, 14, 16].

There is a paucity of data specifically addressing the interplay between CS and OHCA. Cardiac arrest occurring in patients with AMI-related CS confers increased short-term mortality risk with a diminishing effect over time [17]. However, it remains unclear what magnitude of risk for CS development OHCA confers due to the cardiac arrest itself (as opposed to the extent of AMI) and whether these patients have a different prognosis, potentially benefiting from modified treatment options. It is unknown how the interaction between CS and OHCA influences mortality in patients with ST-elevation myocardial infarction (STEMI), who have the largest ischemic myocardial mass.

## Methods

This study aims to provide a national-level analysis of predictors of CS and OHCA in patients with STEMI and their effect on mortality based on an all-comers national registry.

The analysis is based on data from the National Registry of Cardiovascular Surgery and Interventions (NRCSI) in the Czech Republic combined with data from the National Registry of Deaths for mortality analysis.

**Fig. 1:**
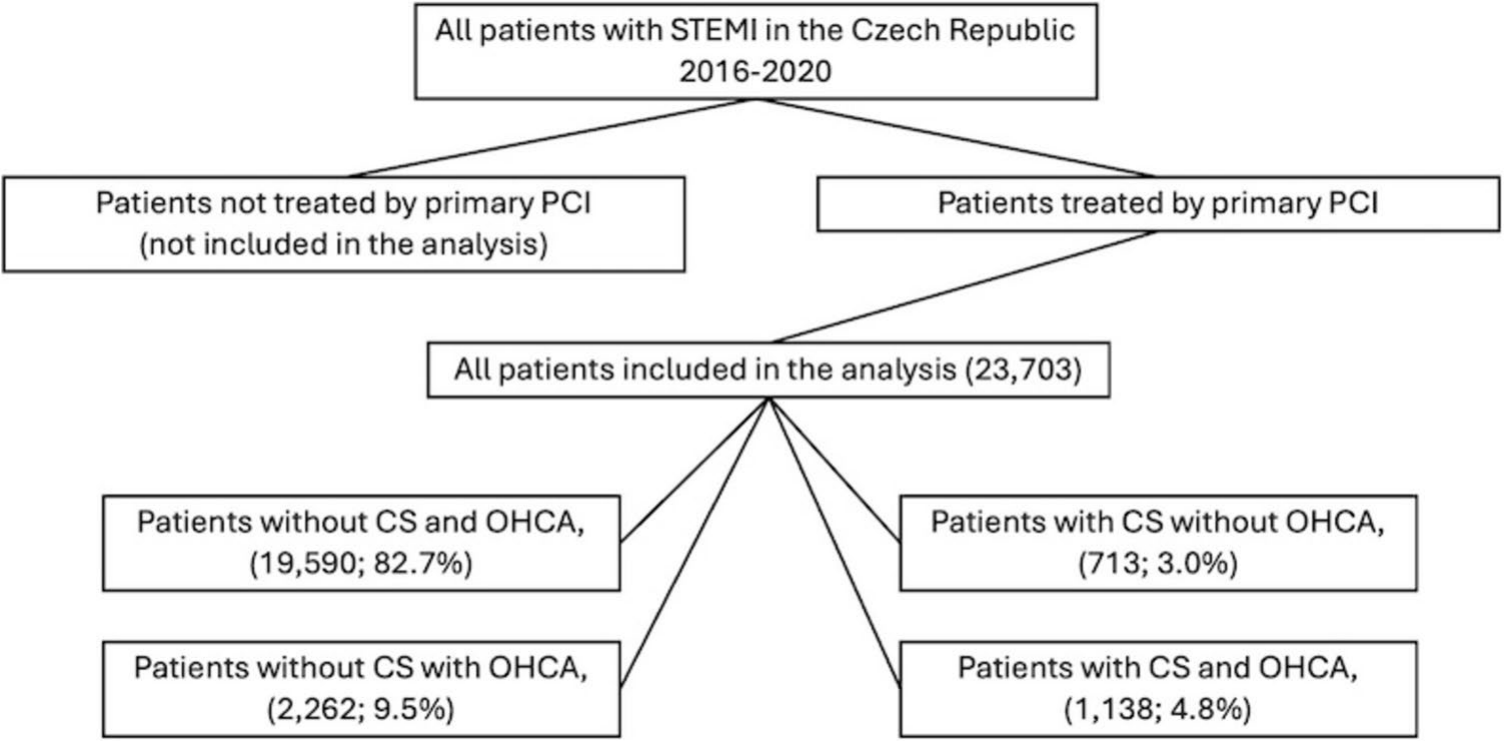
Flowchart of the study

National Health Information System (NHIS) data were collected by the Institute of Health Information and Statistics in the Czech Republic; NHIS provides data on the health status of the population, and on healthcare providers and personnel. The cardiovascular interventions module of the NRCSI started in 2004 and contains mandatory data collected on all coronary and noncoronary catheterizations and cardiovascular interventions performed in the Czech Republic. Data in the NHIS are collected in accordance with Act No. 372/2011 Coll., on Health Services and Conditions of Their Provision. Due to this legal mandate, the retrospective analyses did not require either approval from an ethics committee or informed consent from participants. The investigation conforms to the principles outlined in the *Declaration of Helsinki.* The analysis included all patients with STEMI in the NRCSI in the studied period with no exclusion criteria. Therefore, all the patients included in this study were treated with primary PCI, as patients not undergoing PCI were not included in the NRCSI (Fig. 1). OHCA was defined as any cardiac arrest occurring in the out-of-hospital setting.

STEMI was defined as electrocardiogram changes of ST-segment elevation at the J point in at least two contiguous leads ≥ 0.25 mV in men < 40 years, ≥ 0.2 mV in men ≥ 40 years, or ≥ 0.15 mV in women in leads V2–V3, and/or ≥ 0.1 mV in all other leads.

CS was diagnosed if the patient had a systolic blood pressure < 90 mmHg, or catecholamine use was necessary to maintain a systolic blood pressure ≥ 90 mm Hg, and presented clinical signs of impaired organ perfusion with at least one of the following: altered mental status, cold and clammy skin and limbs, oliguria with urine output < 30 ml per hour, or an arterial lactate level > 2.0 mmol per liter. This definition of CS was used in the design of NRCSI to allow all health care workers entering data into the registry a simple way to differentiate between patients with CS and without CS. Data regarding the medical history, clinical presentation, and PCI were entered into the NRCSI at the time of completion of primary PCI, and both CS and OHCA occurred between the onset of symptoms and the completion of primary PCI.

Standard descriptive statistics were applied in the analysis: absolute and relative frequencies for categorical variables and, for continuous variables, means supplemented with standard deviations and medians with interquartile ranges. The statistical significance of differences between groups of patients was computed using the maximum likelihood Chi-square test for categorical variables and t test for continuous variables. Logistic regression in univariate and multivariate settings with a forward stepwise algorithm applied for identifying the multivariate model was used to analyze factors influencing 30-day and 1-year mortality; its results were described using odds ratios (OR), and their 95% confidence intervals (CI) and statistical significance. Time-to-event data were visualized using the Kaplan–Meier methodology with the landmark analysis approach. The Cox proportional hazards model was adopted for analyzing the combined influences of CS and OHCA in STEMI patients on overall survival described by hazard ratios (HR), and their 95% CI and statistical significance. The level of statistical significance was set at 0.05 for all analyses. Statistical computations were performed using SPSS 27.0.0.0. (IBM Corporation 2022).

## Results

The period from January 1st, 2016, to December 31st, 2020 was chosen for standardized registry data, and 23,703 patients with STEMI were analyzed. The baseline characteristics of the patients are shown in Table 1.

**Table 1:** Baseline characteristics of patients with acute STEMI from the period 2016–2020

		1) CS: no.	2) CS: no	1 vs. 2	3) CS: yes	4) CS: yes	3 vs 4	1 vs 3	2 vs 4	2 vs 3	1 vs 4
		OHCA: no	OHCA: yes	*p*^b^	OHCA: no	OHCA: yes	*p*^b^	*p*^b^	*p*^b^	*p*^b^	*p*^b^
Total^a^		19 590 (100.0%)	2 262 (100.0%)		713 (100.0%)	1 138 (100.0%)					
Gender	Men	13 991 (71.4%)	1 676 (74.1%)	**0.007**	486 (68.2%)	870 (76.4%)	** < 0.001**	0.062	0.134	**0.002**	** < 0.001**
	Women	5 599 (28.6%)	586 (25.9%)		227 (31.8%)	268 (23.6%)					
Age	Mean ± SD	64.0 ± 12.6	62.0 ± 12.6	** < 0.001**	68.8 ± 11.8	64.6 ± 12.3	** < 0.001**	** < 0.001**	** < 0.001**	**0.002**	0.072
	< 40	431 (2.2%)	73 (3.2%)	** < 0.001**	4 (0.6%)	24 (2.1%)	** < 0.001**	** < 0.001**	** < 0.001**	** < 0.001**	0.070
	40–49	2 268 (11.6%)	317 (14.0%)		36 (5.0%)	114 (10.0%)					
	50–59	4 313 (22.0%)	549 (24.3%)		113 (15.8%)	113 (15.8%)					
	60–69	5 930 (30.3%)	677 (29.9%)		212 (29.7%)	212 (29.7%)					
	70–79	4 384 (22.4%)	446 (19.7%)		201 (28.2%)	201 (28.2%)					
	≥ 80	2 264 (11.6%)	200 (8.8%)		147 (20.6%)	147 (20.6%)					
Diabetes mellitus		3 246 (16.6%)	396 (17.5%)	0.260	178 (25.0%)	202 (17.8%)	** < 0.001**	** < 0.001**	0.86	** < 0.001**	0.302
Previous PCI		2 716 (13.9%)	295 (13.0%)	0.279	134 (18.8%)	167 (14.7%)	**0.020**	** < 0.001**	0.192	** < 0.001**	0.446
Previous CABG		524 (2.7%)	65 (2.9%)	0.584	38 (5.3%)	44 (3.9%)	0.140	** < 0.001**	0.126	**0.003**	**0.024**
Chronic kidney disease		536 (2.7%)	49 (2.2%)	0.102	43 (6.0%)	59 (5.2%)	0.440	** < 0.001**	** < 0.001**	** < 0.001**	** < 0.001**
Mechanical ventilation		432 (2.2%)	1 631 (72.1%)	** < 0.001**	194 (27.2%)	1 059 (93.1%)	** < 0.001**	** < 0.001**	** < 0.001**	** < 0.001**	** < 0.001**
Infarct-related artery	LMS	291 (1.5%)	54 (2.4%)	**0.002**	104 (14.6%)	156 (13.7%)	0.597	** < 0.001**	** < 0.001**	** < 0.001**	** < 0.001**
	LAD	8 571 (43.8%)	1 100 (48.6%)	** < 0.001**	366 (51.3%)	611 (53.7%)	0.323	** < 0.001**	**0.005**	0.208	** < 0.001**
	LCX	3 442 (17.6%)	486 (21.5%)	** < 0.001**	163 (22.9%)	279 (24.5%)	0.415	** < 0.001**	**0.047**	0.440	** < 0.001**
	RCA	8 178 (41.7%)	780 (34.5%)	** < 0.001**	229 (32.1%)	309 (27.2%)	**0.023**	** < 0.001**	** < 0.001**	0.243	** < 0.001**
Localization	Anterior	8 380 (42.8%)	1 111 (49.1%)	** < 0.001**	392 (55.0%)	636 (55.9%)	0.702	** < 0.001**	** < 0.001**	**0.006**	** < 0.001**
	Inferior/posterior	9 522 (48.6%)	911 (40.3%)	** < 0.001**	253 (35.5%)	348 (30.6%)	**0.029**	** < 0.001**	** < 0.001**	**0.022**	** < 0.001**
	Lateral	1 494 (7.6%)	206 (9.1%)	0.015	47 (6.6%)	76 (6.7%)	0.942	0.296	**0.015**	**0.031**	0.232
	Not known/LBBB	186 (0.9%)	34 (1.5%)	**0.019**	21 (2.9%)	77 (6.8%)	** < 0.001**	** < 0.001**	** < 0.001**	**0.018**	** < 0.001**
	Unknown	8 (0.0%)	0 (0.0%)	0.186	0 (0.0%)	1 (0.1%)	0.324	0.324	0.335	-	0.512
Time from symptom onset to reperfusion^c^	Median (10–90 perc.)	200 (105; 575)	150 (90; 320)	** < 0.001**	210 (100; 760)	153 (89; 420)	** < 0.001**	0.216	**0.025**	** < 0.001**	** < 0.001**
	Median (IQR)	200 (138; 330)	150 (115; 210)		210 (136; 370)	153 (116; 228)					
	< 2 h (< 120 min)	2 980 (15.4%)	376 (28.4%)	** < 0.001**	119 (17.9%)	290 (26.2%)	** < 0.001**	**0.085**	0.111	** < 0.001**	** < 0.001**
	2-4 h (120–239 min)	8 661 (44.7%)	695 (52.5%)	** < 0.001**	264 (39.7%)	558 (50.4%)	** < 0.001**	**0.011**	0.309	**0.002**	** < 0.001**
	4–8 h (240–479 min)	4 973 (25.7%)	199 (15.0%)	** < 0.001**	161 (24.2%)	170 (15.4%)	** < 0.001**	0.389	0.955	** < 0.001**	** < 0.001**
	> 8 h (≥ 480 min)	2 748 (14.2%)	54 (4.1%)	** < 0.001**	121 (18.2%)	89 (8.0%)	** < 0.001**	**0.005**	** < 0.001**	** < 0.001**	** < 0.001**
Number of diseased vessels	1 VD	9 437 (48.2%)	1 167 (51.6%)	** < 0.001**	181 (25.4%)	374 (32.9%)	** < 0.001**	** < 0.001**	** < 0.001**	** < 0.001**	** < 0.001**
	2 VD	5 719 (29.2%)	648 (28.6%)		215 (30.2%)	332 (29.2%)					
	3 VD	3 552 (18.1%)	415 (18.3%)		270 (37.9%)	396 (34.8%)					
	Not available	882 (4.5%)	32 (1.4%)		47 (6.6%)	36 (3.2%)					
Left main stenosis ˃ 50%		587 (3.0%)	62 (2.7%)	** < 0.001**	108 (15.1%)	163 (14.3%)	0.858	** < 0.001**	** < 0.001**	** < 0.001**	** < 0.001**
TIMI flow before treatment (the lowest value of all treated lesions)	0	10 279 (52.5%)	1 167 (51.6%)	**0.015**	486 (68.2%)	714 (62.7%)	0.096	** < 0.001**	** < 0.001**	** < 0.001**	** < 0.001**
	1	1 663 (8.5%)	186 (8.2%)		71 (10.0%)	120 (10.5%)					
	2	3 403 (17.4%)	453 (20.0%)		80 (11.2%)	159 (14.0%)					
	3	4 245 (21.7%)	456 (20.2%)		76 (10.7%)	145 (12.7%)					
TIMI flow after treatment (the lowest value of all treated lesions)	0	383 (2.0%)	84 (3.7%)	** < 0.001**	61 (8.6%)	82 (7.2%)	**0.021**	** < 0.001**	** < 0.001**	** < 0.001**	** < 0.001**
	1	158 (0.8%)	29 (1.3%)		41 (5.8%)	41 (3.6%)					
	2	866 (4.4%)	120 (5.3%)		90 (12.6%)	116 (10.2%)					
	3	18 183 (92.8%)	2 029 (89.7%)		521 (73.1%)	899 (79.0%)					
MCS											
IABP the same day as PCI		33 (0.2%)	12 (0.5%)	**0.002**	45 (6.3%)	78 (6.9%)	0.647	** < 0.001**	** < 0.001**	** < 0.001**	** < 0.001**
ECMO the same day as PCI		19 (0.1%)	14 (0.6%)	** < 0.001**	21 (2.9%)	87 (7.6%)	** < 0.001**	** < 0.001**	** < 0.001**	** < 0.001**	** < 0.001**
Other MCS the same day as PCI		7 (0.0%)	0 (0.0%)	0.216	5 (0.7%)	10 (0.9%)	0.676	** < 0.001**	** < 0.001**	** < 0.001**	** < 0.001**
IABP from 1 to 30 days after PCI		34 (0.2%)	8 (0.4%)	0.093	8 (1.1%)	7 (0.6%)	0.244	** < 0.001**	0.290	**0.024**	**0.008**
ECMO from 1 to 30 days after PCI		22 (0.1%)	11 (0.5%)	** < 0.001**	6 (0.8%)	21 (1.8%)	0.068	** < 0.001**	** < 0.001**	0.294	** < 0.001**
Other MCS from 1 to 30 days after PCI		14 (0.1%)	3 (0.1%)	0.411	4 (0.6%)	7 (0.6%)	1.000	**0.003**	**0.020**	0.061	** < 0.001**
Mortality at 30 days		776 (4.0%)	239 (10.6%)	** < 0.001**	305 (42.8%)	549 (48.2%)	**0.008**	** < 0.001**	** < 0.001**	** < 0.001**	** < 0.001**
Mortality at 1 year		1 645 (8.4%)	387 (17.1%)	< 0.001	387 (54.3%)	660 (58.0%)	0.116	< 0.001	< 0.001	< 0.001	< 0.001

*CABG* coronary artery bypass graft, *ECMO* extracorporeal membrane oxygenation, *IABP* intra-aortic balloon pump, *LAD* left anterior descending, *LBBB* left bundle branch block, *LCX* left circumflex, *LMS* left main stem, *MCS* mechanical circulatory support, *OHCA* out-of-hospital cardiac arrest, *PCI* percutaneous coronary intervention, *RCA* right coronary artery, *SD* standard deviation, *STEMI* ST-elevation myocardial infarction, *TIMI* thrombolysis in myocardial infarction, *VD* vessel disease

^a^Absolute and relative frequencies for categorical variables; mean supplemented with standard deviation (SD) for age

^b^Pearson Chi-square test for categorical variables; *t* test for age

^c^Computed for patients with available data only

### Patient characteristics

We compared four subgroups of STEMI patients based on the presence of evaluated risk events—OHCA and initial CS: (1) without CS and OHCA (19,590; 82.7%), (2) after OHCA without CS (2,262; 9.5%), (3) with CS without OHCA (713; 3.0%), and (4) after OHCA with CS (1,138; 4.8%). Patients in group 2 had the lowest mean age, 62.0 (± 12.6) years, while patients in group 3 were the oldest, at 68.8 (± 11.8) years. Furthermore, patients in group 3 had the highest proportions of comorbidities, namely diabetes mellitus (DM) (25.0%), chronic kidney disease (CKD) (6.0%), prior PCI (18.8%), and prior coronary artery bypass graft (CABG) (5.3%), which were significantly more common than in all other subgroups with the exception of CKD (5.2%, *p* = 0.44) and prior CABG (3.9%, *p* = 0.14) in group 4. There were fewer comorbidities in groups 1 and 2, and no significant differences were observed between these groups in DM (16.6% vs 17.5%, *p* = 0.26), prior PCI (13.9% vs 13.0%, *p* = 0.279), prior CABG (2.7% vs 2.9%, *p* = 0.584), and CKD (2.7% vs 2.2%, *p* = 0.44). Patients in groups 2 and 4 had a shorter time from symptom onset to reperfusion, with median times (interquartile range, IQR) of 150 (115; 210) min and 153 (116; 228) min, than patients in group 4, who had a median time (IQR) of 210 (136; 370) min, *p* < 0.001, and patients in group 1, with a median time (IQR) of 200 (138; 330) min, *p* < 0.001.

### Coronary angiography

An infarct-related artery (IRA) supplying a large myocardial mass was more common in groups 3 and 4 than in group 1, as was apparent for both the left main stem (LMS) [relative risk (RR) 9.82, 95% CI 7.95;12.13, *p* < 0.001 in group 3; RR 9.23, 95% CI 7.67;11.10, *p* < 0.001 in group 4] and the left anterior descending artery (LAD) (RR 1.17, 95% CI 1.09;1.26, *p* < 0.001 in group 3; RR 1.23, 95% CI 1.16;1.30, *p* < 0.001 in group 4). The relationship with supplied myocardial territory was less pronounced, but still significant in group 2 (RR 1.61, 95% CI 1.21;2.14, *p* < 0.001 for LMS; RR 1.11, 95% CI 1.06;1.16, *p* < 0.001 for LAD). The distribution of IRAs among groups translated into ST-elevation localization, with anterior STEMI being less frequent in group 1 (42.8%) and with increasing risk in group 2 (RR 1.15, 95% CI 1.10;1.20, *p* < 0.001), group 3 (RR 1.29, 95% CI 1.20;1.38, *p* < 0.001), and group 4 (RR 1.31, 95% CI 1.24;1.38, *p* < 0.001). The presence of triple-vessel disease was also less common in group 1 (18.1%), similar to group 2 (RR 1.01, 95% CI 0.92;1.11, *p* = 0.80), and more common in group 3 (RR 1.92, 95% CI 1.76;2.09, *p* < 0.001) and group 4 (RR 2.09, 95% CI 1.89;2.31, *p* < 0.001). Groups 3 and 4 had lower initial as well as final thrombolysis in myocardial infarction (TIMI) flow in the IRA than groups 1 and 2 (*p* < 0.001).

### Mechanical ventilation and circulatory support

The use of mechanical ventilation was lowest in group 1 (2.2%), and the risk of ventilation support needs increased in group 3 (RR 12.34; 95% CI 10.60;14.36, *p* < 0.001), group 2 (RR 32.70, 95% CI 29.68;36.02, *p* < 0.001), and group 4 (RR 42.20; 95% CI 38.39;46.39, *p* < 0.001). The CS status played a role in the use of mechanical circulatory support (MCS) devices. This was evident for intra-aortic balloon pump (IABP) use on the day of primary PCI, which was least frequent in group 1 (0.2%) and increased in group 2 (RR 3.15; 95% CI 1.62;6.09, *p* = 0.001), group 3 (RR 37.47, 95% CI 24.06;58.35, *p* < 0.001), and group 4 (RR 40.69, 95% CI 27.20;60.86, *p* < 0.001), and even more in extracorporeal membrane oxygenation (ECMO) use on the day of primary PCI, which was again least common in group 1 (0.1%) and increased in group 2 (RR 6.38; 95% CI 3.20;12.71, *p* < 0.001), group 3 (RR 30.38, 95% CI 16.41;56.26, *p* < 0.001), and group 4 (RR 78.86, 95% CI 48.18;129.08, *p* < 0.001).

### Outcome

The presence of CS played a role in mortality. Additionally, the highest 30-day mortality rate was in the subgroup combining both studied risks, group 4 (48.2%), followed by group 3 (42.8%), group 2 (10.6%), and lowest in group 1 (4.0%). The predictors of 30-day mortality in the multivariate logistic model based on a forward stepwise algorithm are shown in Table 2 for patients after OHCA and in Table 3 for patients without OHCA. CS was a predictor of 30-day mortality with an OR (95% CI) of 5.53 (4.51; 6.75) for patients after OHCA and an OR (95% CI) of 9.28 (7.56; 11.38) for those without OHCA. Other independent predictors of 30-day mortality for all patients were age, DM, need for mechanical ventilation, IRA, multivessel disease, final TIMI flow, and the utilization of MCS. A further predictor for patients without OHCA was CKD. In addition, CS remained a predictor of 1-year mortality, with an OR (95% CI) of 4.66 (3.87; 5.61) for patients after OHCA and an OR (95% CI) of 7.33 (6.04; 8.89) for those without OHCA. The predictors of 1-year mortality in the multivariate logistic model based on a forward stepwise algorithm are shown in Table 4 for patients after OHCA and in Table 5 for patients without OHCA.

**Table 2:** Predictors of 30-day mortality in patients with acute STEMI after OHCA (multivariate model)

Characteristics^a^	OR (95% CI)	*P*
Cardiogenic shock	5.52 (4.51; 6.75)	< 0.001
Age at intervention		
< 40	Reference	
40–49	0.77 (0.36; 1.66)	0.510
50–59	0.83 (0.40; 1.72)	0.619
60–69	1.75 (0.86; 3.56)	0.122
70–79	3.13 (1.53; 6.43)	0.002
≥ 80	4.91 (2.35; 10.27)	< 0.001
Diabetes mellitus	1.37 (1.08; 1.73)	0.010
Mechanical ventilation	1.89 (1.40; 2.55)	< 0.001
LMS	2.14 (1.52; 3.02)	< 0.001
Number of diseased vessels		
1 VD	Reference	
2 VD	1.12 (0.88; 1.42)	0.368
3 VD	1.46 (1.15; 1.86)	0.002
Not available	1.84 (1.01; 3.37)	0.047
TIMI flow after procedure 0–2	2.49 (1.96; 3.16)	< 0.001
IABP the same day as PCI	1.95 (1.18; 3.21)	0.009
ECMO the same day as PCI	3.78 (2.36; 6.05)	< 0.001
IABP from 1 to 30 days after PCI	4.28 (1.30; 14.12)	0.017
ECMO from 1 to 30 days after PCI	4.43 (1.83; 10.67)	< 0.001
Other MCS from 1 to 30 days after PCI	0.12 (0.02; 0.78)	0.026

*CI* confidence interval, *ECMO* extracorporeal membrane oxygenation, *IABP* intra-aortic balloon pump, *LMS* left main stem, *MCS* mechanical circulatory support, *OHCA* out-of-hospital cardiac arrest, *OR* odds ratio, *STEMI* ST-elevation myocardial infarction, *TIMI* thrombolysis in myocardial infarction, *VD* vessel disease

^a^Model is based on forward selection algorithm with cardiogenic shock forced into the model

**Table 3:** Predictors of 30-day mortality in patients with acute STEMI without OHCA (multivariate model)

Characteristics^a^	OR (95% CI)	*P*
Cardiogenic shock	9.28 (7.56; 11.38)	< 0.001
Age at intervention		
< 40	Reference	
40–49	1.03 (0.30; 3.53)	0.959
50–59	1.38 (0.43; 4.46)	0.589
60–69	3.73 (1.18; 11.78)	0.025
70–79	6.49 (2.05; 20.48)	0.001
≥ 80	17.03 (5.40; 53.75)	< 0.001
Diabetes mellitus	1.27 (1.08; 1.49)	0.004
Chronic kidney disease	1.56 (1.18; 2.08)	0.002
Mechanical ventilation	2.38 (1.84; 3.08)	< 0.001
LCX	0.75 (0.63; 0.91)	0.003
RCA	0.56 (0.48; 0.65)	< 0.001
Number of diseased vessels		
1 VD	Reference	
2 VD	1.34 (1.12; 1.60)	0.001
3 VD	1.85 (1.55; 2.22)	< 0.001
Not available	1.31 (0.93; 1.86)	0.125
LMS stenosis ˃ 50%	1.13 (1.07; 1.18)	< 0.001
TIMI flow after procedure 0–2	3.29 (2.77; 3.89)	< 0.001
IABP the same day as PCI	3.27 (1.84; 5.81)	< 0.001
ECMO the same day as PCI	5.30 (2.43; 11.59)	< 0.001
IABP from 1 to 30 days after PCI	16.70 (8.24; 33.86)	< 0.001
ECMO from 1 to 30 days after PCI	17.95 (6.97; 46.22)	< 0.001
Other MCS from 1 to 30 days after PCI	6.04 (0.98; 37.09)	0.052

*CI* confidence interval, *ECMO* extracorporeal membrane oxygenation, *IABP* intra-aortic balloon pump, *LCX* left circumflex, *LMS* left main stem, *MCS* mechanical circulatory support, *OHCA* out-of-hospital cardiac arrest, *OR* odds ratio, *RCA* right coronary artery, *STEMI* ST-elevation myocardial infarction, *TIMI* thrombolysis in myocardial infarction, *VD* vessel disease

^a^Model is based on forward selection algorithm with cardiogenic shock forced into the model

**Table 4:** Predictors of 1-year mortality in patients with acute STEMI after OHCA (multivariate model)

Characteristics^a^	OR (95% CI)	*p*
Cardiogenic shock	4.66 (3.87; 5.61)	< 0.001
Age at intervention		
< 40	Reference	
40–49	0.75 (0.37; 1.49)	0.406
50–59	1.11 (0.58; 2.14)	0.757
60–69	1.92 (1.01; 3.66)	0.048
70–79	3.53 (1.83; 6.80)	< 0.001
≥ 80	5.82 (2.96; 11.46)	< 0.001
Diabetes mellitus	1.44 (1.15; 1.80)	0.002
Chronic kidney disease	1.67 (1.04; 2.67)	0.033
Mechanical ventilation	2.03 (1.56; 2.63)	< 0.001
LMS	2.05 (1.44; 2.91)	< 0.001
Number of diseased vessels		
1 VD	Reference	
2 VD	1.32 (1.06; 1.63)	0.012
3 VD	1.78 (1.43; 2.22)	< 0.001
Not available	2.18 (1.21; 3.92)	0.009
TIMI flow after procedure 0–2	1.88 (1.49; 2.38)	< 0.001
IABP the same day as PCI	2.41 (1.40; 4.15)	0.002
ECMO the same day as PCI	4.15 (2.50; 6.89)	< 0.001
IABP from 1 to 30 days after PCI	5.55 (1.59; 19.41)	0.007
ECMO from 1 to 30 days after PCI	3.96 (1.66; 9.45)	0.002

*CI* confidence interval, *ECMO* extracorporeal membrane oxygenation, *IABP* intra-aortic balloon pump, *LMS* left main stem, *OHCA* out-of-hospital cardiac arrest, OR odds ratio, *STEMI* ST-elevation myocardial infarction, *TIMI* thrombolysis in myocardial infarction, *VD* vessel disease

^a^Model is based on forward selection algorithm with cardiogenic shock forced into the model

**Table 5:** Predictors of 1-year mortality in patients with acute STEMI without OHCA (multivariate model)

Characteristics^a^	OR (95% CI)	*P*
Cardiogenic shock	7.33 (6.04; 8.89)	< 0.001
Age at intervention		
< 40	Reference	
40–49	0.99 (0.44; 2.25)	0.980
50–59	1.43 (0.66; 3.12)	0.366
60–69	3.45 (1.61; 7.42)	0.001
70–79	7.01 (3.27; 15.04)	< 0.001
≥ 80	19.53 (9.11; 41.90)	< 0.001
Diabetes mellitus	1.36 (1.20; 1.54)	< 0.001
Previous PCI	1.38 (1.20; 1.58)	< 0.001
Chronic kidney disease	2.10 (1.69; 2.60)	< 0.001
Mechanical ventilation	2.17 (1.73; 2.72)	< 0.001
LMS	1.57 (1.18; 2.08)	0.002
LAD	1.21 (1.05; 1.40)	0.007
RCA	0.85 (0.74; 0.99)	0.036
Number of diseased vessels		
1 VD	Reference	
2 VD	1.13 (1.00; 1.29)	0.056
3 VD	1.55 (1.36; 1.77)	< 0.001
Not available	1.27 (0.98; 1.64)	0.071
LMS stenosis ˃ 50%	1.07 (1.02; 1.11)	0.005
TIMI flow after procedure 0–2	2.34 (2.02; 2.70)	< 0.001
IABP the same day as PCI	4.65 (2.61; 8.28)	< 0.001
ECMO the same day as PCI	5.47 (2.45; 12.18)	< 0.001
IABP from 1 to 30 days after PCI	19.45 (9.09; 41.64)	< 0.001
ECMO from 1 to 30 days after PCI	20.20 (8.15; 50.07)	< 0.001

^a^Model is based on forward selection algorithm with cardiogenic shock forced into the model

*CI* confidence interval, *ECMO* extracorporeal membrane oxygenation, *IABP* intra-aortic balloon pump, *LCX* left circumflex, *LMS* left main stem, *OHCA* out-of-hospital cardiac arrest, *OR* odds ratio, *RCA* right coronary artery, *STEMI* ST-elevation myocardial infarction, *TIMI* thrombolysis in myocardial infarction, *VD* vessel disease

**Fig. 2:**
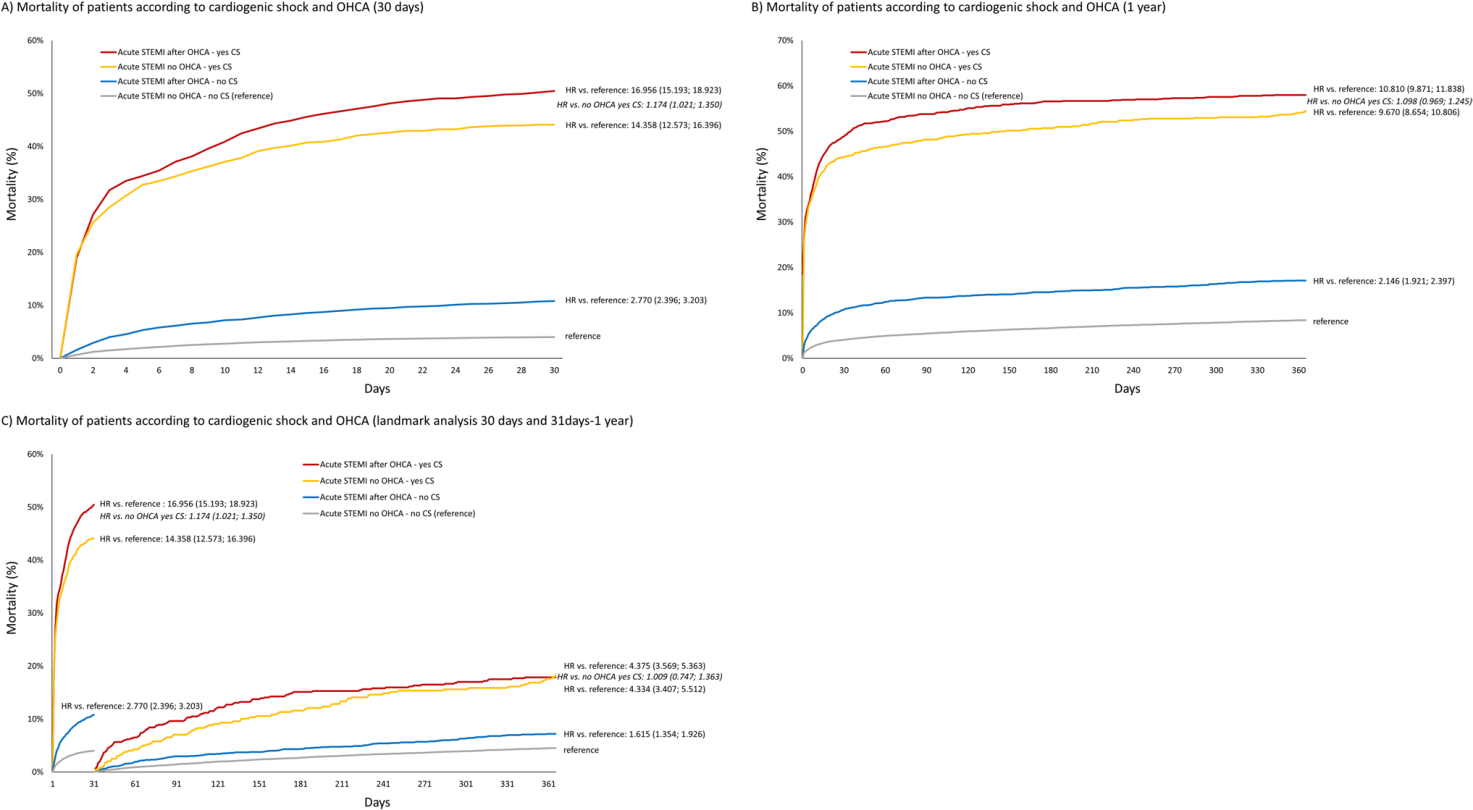
**A** Mortality of patients according to cardiogenic shock and out-of-hospital cardiac arrest (30 days). **B** Mortality of patients according to cardiogenic shock and out-of-hospital cardiac arrest (1 year). **C** Mortality of patients according to cardiogenic shock and out-of-hospital cardiac arrest (landmark analysis 30 days and 31 days–1 year)

Figure 2 shows the Kaplan–Meier mortality curves of all compared patient groups. For overall survival up to 30 days in comparison to group 1, patients in group 2 had an HR (95% CI) of 2.77 (2.40; 3.20), patients in group 3 an HR (95% CI) of 14.36 (12.57; 16.40), and patients in group 4 an HR (95% CI) of 16.96 (15.19; 18.92); OHCA significantly modified the 30-day survival in both patients with CS [HR (95% CI) of 1.17 (1.02; 1.35)] and patients without CS [HR (95% CI) of 2.77 (2.40; 3.20)]. This data shows a significant and combined effect of CS and OHCA on mortality, which lasted to a lesser extent even in patients who survived for the first 30 days. Compared to patients in group 1, patients in group 2 had an HR (95% CI) of 1.62 (1.35; 1.93), patients group 3 an HR (95% CI) of 4.33 (3.41; 5.51), and patients in group 4 an HR (95% CI) of 4.38 (3.57; 5.36) for overall survival up to 1 year. The modifying effect of OHCA remained significant only in patients without CS [HR (95% CI) of 1.62 (1.35; 1.93)] and diminished in those with CS [HR (95% CI) of 1.01 (0.75; 1.36)].

## Discussion

Our analysis of patients with acute STEMI treated with primary PCI revealed differences between populations depending on their CS and OHCA status, as well as the impact of these risk conditions on patient prognosis.

Both CS and OHCA are complications of AMI that result in a markedly worse prognosis. The occurrence of CS among AMI patients is reported between 5 and 10% [3, 7, 11]. Our data showed a 7.8% prevalence of CS among patients with STEMI in an all-comers national registry, which is consistent with previous studies from other regions [3, 7, 11]. CS is a well-known predictor of mortality in AMI patients—RCTs commonly report 30-day mortality rates between 40 and 60% [5, 9, 10, 12]. CS also remains the major cause of in-hospital mortality in AMI patients [18]. The 30-day mortality rate for CS patients in our study was 42.8% in the subgroup without OHCA and 48.2% in cases complicated by OHCA. Chronic comorbidities, namely prior myocardial infarction, heart failure, DM, and CKD, in CS patients who present with AMI often lead to more severe coronary artery disease (CAD) and lower functional reserves and are associated with increased mortality [19, 20]. Our data confirmed the role of DM as an independent predictor of worse prognosis in STEMI patients, while the association with CKD was significant only in patients without OHCA.

The extent of CAD at the time of presentation is a logical predictor of severe myocardial ischemia. This relationship is most apparent in the presence of chronic total occlusion (CTO), which is supplied via collaterals from the IRA. Acute occlusion of the IRA then leads to double jeopardy of both myocardial territories, a greater risk of CS occurrence, and a worse prognosis [21]. While our registry did not include the presence of CTOs in our patients, the severity of CAD, as represented by multivessel disease and LMS disease, was associated with increased mortality. This association was again more pronounced in patients without OHCA.

OHCA complicating AMI in patients treated with primary PCI has been reported in 5–30% of patients [13–16]. There is variability in reporting outcome predictors in studies of CS complicating AMI [22]. As an example, for cardiac arrest—one of the most frequently reported prognostic factors—80% of RCTs report the condition in patients with CS after AMI; however, the prevalence ranging from 0 to 92% [22] highlights the heterogeneity of the studied populations. The all-comers national registry with the recorded resuscitation status of patients with STEMI treated with primary PCI depicts a reliable prevalence of OHCA in these patients, although it is modified by prehospital care for patients with OHCA and the rules for termination of resuscitation. In the Czech Republic, Emergency Medical Services have the authority to terminate resuscitation attempts outside the hospital, thereby conducting a selection process for patients with OHCA admitted to the catheterization laboratory. The prevalence of OHCA was 14.3% in our STEMI population and 61.5% in patients with CS. One of the factors that could influence the reported prevalence of OHCA in patients with AMI treated with primary PCI in various regions is the availability and use of extracorporeal cardiopulmonary resuscitation (ECPR) [13]. While ECPR use in the Czech Republic is on the rise, including a local RCT on this topic [23], there are significant differences in the transport time to catheterization laboratories in urban and rural areas, and the effective use of ECPR remains limited to major cities. Thus, the national-level prevalence of OHCA in patients with STEMI may differ from that in specific settings of prehospital care for cardiac arrest in large cities [13].

The complex interplay between OHCA and CS within the STEMI population results in intriguing clinical and demographic distinctions among the studied subgroups. Our patients with CS and without OHCA combined the highest baseline risk represented by older age, extent of chronic CAD, and comorbidities with a high risk of the acute ischemic insult represented by an IRA supplying a large myocardial mass. On the other hand, patients with OHCA without CS were the youngest, with fewer comorbidities and smaller acute ischemic insult than patients with CS. Nevertheless, the myocardial territory at risk during AMI was still larger, and their comorbidities, despite younger age, were similar to those of patients without OHCA and CS. Therefore, these patients may represent a particularly vulnerable population among the STEMI population. We found the worst prognosis in patients with both CS and OHCA. This subgroup combined younger age, intermediate level of comorbidities, and high acute ischemic risk, suggesting that while OHCA occurs in younger and healthier patients, a large ischemic myocardial mass is the most important factor in CS development.

OHCA increased the 30-day mortality of our patients to a similar absolute risk difference in both the CS and non-CS subgroups, but the relative risk difference was obviously much greater in the non-CS subgroup, where OHCA more than doubled the risk of 30-day mortality after STEMI. The effect of OHCA on the mortality of patients with CS was lower than previously reported, while the prevalence of OHCA in our patients with CS was slightly greater [17]. Combined with findings that patients with AMI after OHCA most commonly die due to neurological injury and not primary cardiac reasons [6], the different effect of OHCA on mortality in the CS population may reflect the heterogeneity of OHCA in various settings. We did not report the time to return of spontaneous circulation or specific cause of death in our registry, but the studied population included patients with low or no neurological impact of OHCA, as is perhaps best illustrated by the 72% prevalence of mechanical ventilation in patients with OHCA and without CS. Nevertheless, OHCA proved to be an important predictor of 30-day mortality in patients with STEMI, which was observed across the whole studied population with similar effect. Due to the clinical presentation, OHCA led to a significantly decreased time from symptom onset to reperfusion in patients with STEMI, but this reduction did not outweigh the deleterious effect of OHCA on survival.

The impact of OHCA on mortality in our analysis diminished over time and became statistically nonsignificant at 1 year among patients with CS. A similar effect has been reported previously and suggests that both baseline characteristics and the extent of ischemic insult during AMI in patients with OHCA influence the prognosis for a shorter duration than in patients with CS [17].

The limitations of our study are primarily inherent to its retrospective and observational design. In the national-level analysis, the number of collected variables was limited and we cannot rule out the possibility of an undetected confounding factor as we were unable to analyze all the granular patient-level data. The absence of time to return of spontaneous circulation in our dataset represents a possible bias, as it does not allow the assessment of OHCA duration on the prognosis of patients with STEMI. The shorter times from symptom onset to reperfusion in patients with OHCA may represent a survivor bias and attenuate the effects of OHCA on patient’s mortality rates. The reduction of total ischemic time in these patients were probably due to the reduction in times from symptom onset to hospital admission, but these variables were not included in our study. The simplified definition of CS may lead to a bias, as patients with other types of shock fulfill this definition as well. Inclusion of patients with septic or hemorrhagic shock into the studied population may affect the results of our analysis. However, the combination of shock, STEMI, and coronary artery lesions treated with primary PCI in all our patients with CS suggests that most of these patients were, in fact, suffering from CS. Due to the nature of NRCSI, only patients with STEMI treated with primary PCI could be included in our study. This represents a selection bias, as patients with STEMI not treated with PCI due to any reasons were not considered in our analysis, thus limiting the generalizability of the results to all patients with STEMI. The effect of COVID-19 pandemic on the results of our analysis was not studied and the pandemic may have had a specific effect on the prognosis of patients in the last year of the studied period.

In addition, the analysis was performed on a homogenous population, and its conclusions may not be generalizable to other populations. We specifically focused on patients with STEMI treated with primary PCI, and the results may differ from those of the broader population of patients with AMI. Further studies are needed to address these topics.

## Conclusion

Our national-level analysis based on all-comers registry of patients with STEMI showed that OHCA significantly altered the 30-day mortality risk for both patients with and without CS. At 1 year, OHCA altered the mortality risk only for patients without CS. CS is a strong predictor of both 30-day and 1-year mortality in STEMI, irrespective of OHCA. Patients with STEMI after OHCA and CS at admission are at the highest risk of death. Patients with CS had the oldest age, the highest proportion of comorbidities, and the highest extent of CAD, while patients with OHCA had the youngest age, but an increased extent of CAD.

## Data Availability

The datasets analyzed during the current study are not publicly available due to individual privacy, but are available from the corresponding author on reasonable request.
